# Micromagnetic Investigation on Microstructure Modulation and Magnetic Properties of Nd-Fe-B Permanent Magnets

**DOI:** 10.3390/nano16080460

**Published:** 2026-04-14

**Authors:** Lingbo Bao, Hargen Yibole, Guohong Yun, Bai Narsu, Yongjun Cao, Hui Yang, Jiaqi Fu, Ruotong Zhang

**Affiliations:** 1 College of Artificial Intelligence, Inner Mongolia Normal University, 81 Zhaowuda Rd., Hohhot 010022, China; 2 College of Physics and Electronic Information, Inner Mongolia Key Laboratory of Applied Condensed Matter Physics, Inner Mongolia Normal University, 81 Zhaowuda Rd., Hohhot 010022, China

**Keywords:** Nd-Fe-B permanent magnets, micromagnetics, Hybrid Monte Carlo, grain boundary, coercivity

## Abstract

The magnetic properties of materials similar to Nd-Fe-B permanent magnets are highly sensitive to microstructure. Using Hybrid Monte Carlo micromagnetics simulations, we systematically investigate how grain boundary (GB) and grain crystallographic orientation affect coercivity (*H_c_*) and remanence (*M_r_*). A polycrystalline model with independently adjustable microstructural parameters is constructed via Voronoi tessellation. Our results show that increasing GB width from 2 nm to 10 nm reduces *Hc* by 32% and *M_r_* by 16%. Grain boundary acts as both a nucleation site and pinning center: a wider GB facilitates reverse domain nucleation, especially at the triple junctions. However, domain wall propagation is underpinned by GB during the propagation process. For a thick GB, *H_c_* decreases with increasing GB saturation magnetization (*M_s_*′), because the thick weakly magnetic layer weakens exchange coupling between adjacent grains, shifting the reversal behavior from collective switching to more localized nucleation. Increasing the average easy-axis tilt angle reduces *H_c_*, as the misalignment lowers the effective anisotropy component along the applied field direction, facilitating magnetization reversal. These findings confirm the importance of GB and texture control in optimizing the magnetic performance of Nd-Fe-B permanent magnets, offering references for experimental investigations.

## 1. Introduction

Nd-Fe-B permanent magnets have become indispensable in modern technologies, from electric vehicles to wind turbines, due to their exceptional magnetic properties [1,2]. Since their discovery nearly four decades ago, significant efforts have been devoted to understanding and improving their performance. The high coercivity of these magnets originates from the large magnetocrystalline anisotropy of the Nd_2_Fe_14_B phase, with K_1_ ≈ 4.5 × 10^7^ erg/cm^3^ at room temperature [3]. However, the magnetic properties of Nd-Fe-B magnets are remarkably sensitive to their microstructure [4,5]. Experimental work has shown that the grain boundary (GB) phase plays a crucial role. Its width, composition, and magnetic properties directly influence both coercivity and remanence [6,7,8,9,10,11]. For example, the GB diffusion of heavy rare earth elements such as Dy can substantially enhance coercivity by creating high-anisotropy shells around the Nd_2_Fe_14_B grains [12,13]. Grain size or composition [14,15,16], crystallographic alignment [17], and grain shape [16,18] also affect magnetization reversal, but their effects are often intertwined in experiments.

Micromagnetics simulations offer a way to disentangle these factors by independently controlling each microstructural parameter [19,20,21]. The Hybrid Monte Carlo (HMC) method is particularly well-suited for studying temperature-dependent micromagnetic problems as it samples magnetic configurations according to the Boltzmann distribution [22,23].

In this work, we use HMC simulations to systematically investigate how GB properties and crystallographic orientation affect the magnetic behavior of Nd-Fe-B magnets. We construct a polycrystalline model with adjustable microstructural features and examine their effects on coercivity, remanence, and magnetization reversal. Our aim is to provide clear, quantitative guidelines for microstructure optimization.

## 2. Model and Methods

### 2.1. Hybrid Monte Carlo Micromagnetics

We employed the HMC method, which was originally developed for finite-temperature micromagnetics simulations. This method is a numerical simulation approach widely used in lattice field theory, which transforms deterministic problems into stochastic ones, and its primary purpose is to generate a Boltzmann-like distribution [22]. The detailed implementation and validation of the HMC method for magnetic materials have been reported in our previous work [24,25].

For HMC micromagnetics, a momentum ***Π_i_*** is introduced for the magnetization ***M_i_*** in the ith cell, as a pair of conjugate variables as the position and momentum in mechanics. The momentum *Π_i_* is generated following a Gaussian distribution exp($-V_{c}\sum_{i}{\overset{⃑}{\Pi }}_{i}^{2}/2k_{B}T$). The correct Boltzmann distribution with respect to the free energy exp(−*F*{***M_i_***}/*k*_B_*T*) is obtained by simulations divided into successive trajectories. Within each trajectory, the {***M_i_***, ***Π_i_***} pairs are iterated following the Hamilton equations; then, the Monte Carlo judgment is used to accept or reject the new distribution of {***M_i_***} at the end of each trajectory. The magnetic free energy density *F*/*V*_c_ has the following terms [22]:

(1) $$\frac{F}{V_{C}}=E_{ext}+E_{ex}+E_{m}+E_{ani}$$

The total free energy of the system consists of four contributions: the Zeeman energy, *Eext*; exchange energy, *Eex*; magnetostatic energy, *Em*; and magnetocrystalline anisotropy energy, *Eani*.

(2) $$E_{ext}=-\sum_{i}H_{ext}\cdot M_{i}$$

(3) $$E_{ex}=\frac{{A^{*}}_{ij}^{2}}{M_{s}^{2}D^{2}}{\sum_{i}\sum_{j}\left(M_{i}-M_{j}\right)}^{2}$$

(4) $$E_{m}=2\pi \sum_{i}\sum_{j}M_{i}\tilde{N_{ij}}M_{j}.$$

(5) $$E_{ani}=-\sum_{i}K{\left(M_{i}\cdot k_{c}\right)}^{2}+\frac{bK}{4}{\left(M_{i}^{2}-M_{s}^{2}\right)}^{2}$$

*V*_c_ is the cell volume. ${\tilde{N}}_{ij}$ is the demagnetizing matrix; $M_{s}$ is the saturation magnetization; *K* is the anisotropy constant (uniaxial anisotropy is assumed at 300 K); and A* is the exchange constant. The term $\frac{bK}{4}{\left(M_{i}^{2}-M_{s}^{2}\right)}^{2}inEani$ is the constraint potential used in HMC simulation. *b* is a dimensionless parameter of double-well constraint potential, which in this paper is *b* = 10.

### 2.2. Polycrystalline Model Construction

**Figure 1:**
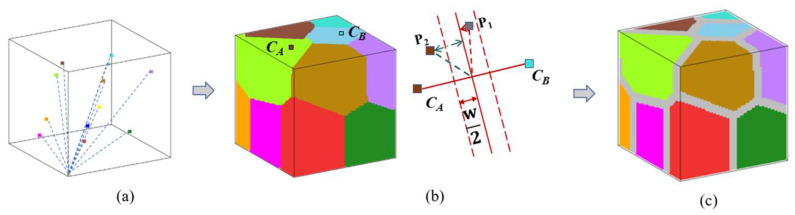
(**a**) Randomly distributed grain centers (each color corresponds to one grain) and Voronoi tessellation assigning cells to grains. (**b**) Diagram of method for dividing the grain boundary. (**c**) A complete polycrystal model with the grain boundary and grains.

As shown in Figure 1, we constructed polycrystalline models using Voronoi tessellation [26]. The simulation volume is 128 × 128 × 128 nm^3^, discretized into 64 × 64 × 64 cubic cells of 2 nm each (The Néel exchange length $\delta_{ex}={\left(A^{*}/2\pi M_{s}^{2}\right)}^{1/2}$ is about 2.758 nm). Periodic boundary conditions were applied.

Once the model construction method is established, an arbitrary number of “seeds” can be distributed in the volume to obtain polycrystalline models with any desired number of grains, as shown in Figure 1a. Under the same dimensions, a larger number of seeds results in a smaller average grain size, which can be used to simulate the effect of grain size on the magnetic properties of the material. Using the same approach, polycrystalline models with different grain shapes can also be constructed to study the effect of grain shape on the magnetic properties of the material.

The GB is defined as shown in Figure 1b. For two adjacent grain centers, *C_A_* and *C_B_*, the cell P_1_ is assigned to the GB if its distance to the perpendicular bisector of the line *C_A_C_B_* is less than half the desired GB width *w*. Otherwise, it remains part of the grain, as in point P_2_. By iterating over every cell, we can label all GB, as shown in Figure 1c.

### 2.3. Material Parameters

Table 1 lists the magnetic parameters for Nd_2_Fe_14_B at temperature *T* = 300 K used in our simulations, where *α_θ_* is a dimensionless constant for tuning the anisotropic texture. The crystallographic orientation of each grain is described by a tilt angle *θ* relative to the global *z*-axis. The distribution of *θ* follows the von Mises distribution [17], which is then normalized by adjusting the coefficient *c*_1_. The azimuthal angle is random within the range of 0 to 2π.

(6) $$f\left(\theta \right)=c_{1}\exp\left(-\alpha_{\theta }\sin\theta^{2}\right)$$

(7) $$1=c_{1}\int d\theta f\left(\theta \right)\;\;\;\;\;\;\;\theta \in \left[0,\pi \right]$$

After applying the half-angle formula, by setting $\theta ’=2\theta ,c_{2}={c_{1}e}^{\left(-\alpha_{\theta }/2\right)}$, Equation (6) can be transformed into

(8) $$f\left(\theta^{\prime }\right)=c_{2}\exp\left(\frac{\alpha_{\theta }}{2}\cos\theta^{\prime }\right)$$

(9) $$1=c_{2}\int d\theta^{\prime }f\left(\theta^{\prime }\right)\;\;\;\;\;\;\;\;\theta \in \left[0,\pi /2\right]$$

Table 2 presents the average angles $\bar{\theta }$ of the easy-axes of each grain corresponding to different *c*_1_ when the number of grains (*N*) is 24. During the calculation, to eliminate the influence of orientation, a relatively large value is adopted so the material is oriented along the *z*-axis, and then the influence of other parameters is qualitatively analyzed.

**Figure 2:**
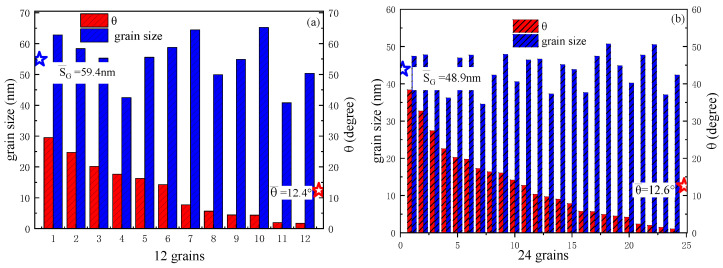

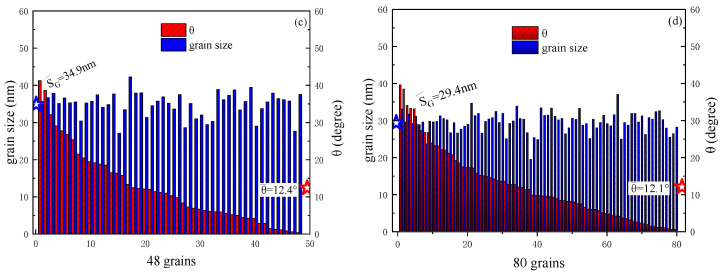
Histogram distributions of the easy-axis tilt angles (red columnar) and the grain size (blue columnar) of each grain with different grain numbers: (**a**) *N* = 12; (**b**) *N* = 24; (**c**) *N* = 48; (**d**) *N* = 80. The blue stars represent the average grain size, and the red stars represent the average tilt angles.

The grain size obtained by Voronoi partitioning is relatively uniform. Figure 2 shows statistics for the $\bar{\theta }$ and average grain size ($\bar{S_{G}}$) for different numbers of grains. The blue-colored sections in the histogram of Figure 2 show that when the number of grains is 12, the $\bar{S_{G}}$ is 59.4 nm; when the number of grains is 80, the $\bar{S_{G}}$ is 29.4 nm. For the distribution of tilt angles, however, increasing the number of grains does not significantly change the $\bar{\theta }$ (red star points).

**Figure 3:**
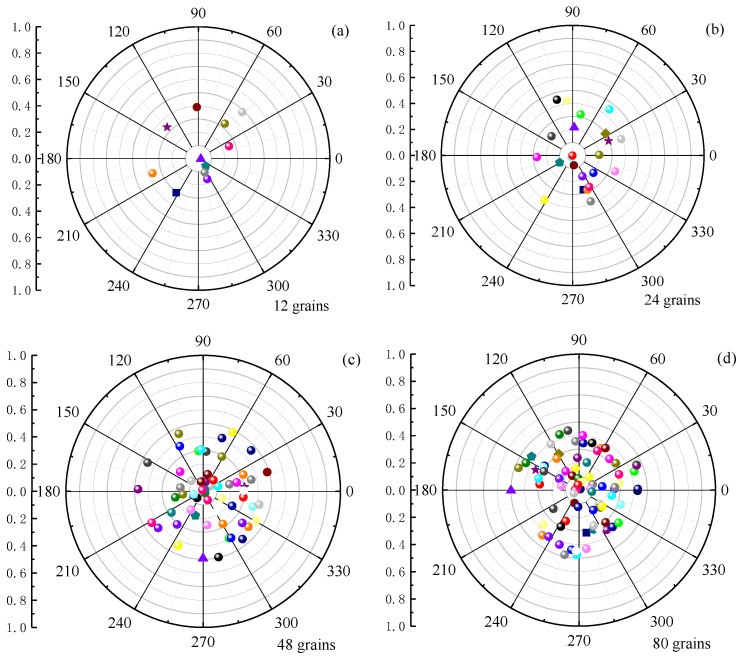
Distributions of the easy-axis tilt angles in polar coordinates with (**a**) *N* = 12, (**b**) *N* = 24, (**c**) *N* = 48, and (**d**) *N* = 80. Different colors represent the tilt angles of grains.

To visually observe the orientation of the grain easy axes, we plotted the distribution of each grain’s easy-axis direction relative to the *z*-axis (pointing out of the page) in polar coordinates for different numbers of grains. As can be seen from Figure 3, *θ* is confined to within 40° according to the distribution, while the azimuthal angle is completely random. As the number of grains increases, the number of data points within 40° increases, and the tilt angles between adjacent grains become increasingly closer. For example, for 80 grains, the $\bar{\theta }$ between adjacent grains is within 1°, whereas for 12 grains, the $\bar{\theta }$ between adjacent grains is about 4°. This indicates that we can arbitrarily regulate the grain size and anisotropic orientation.

## 3. Results and Discussion

### 3.1. Effect of Grain Boundary Width

**Figure 4:**
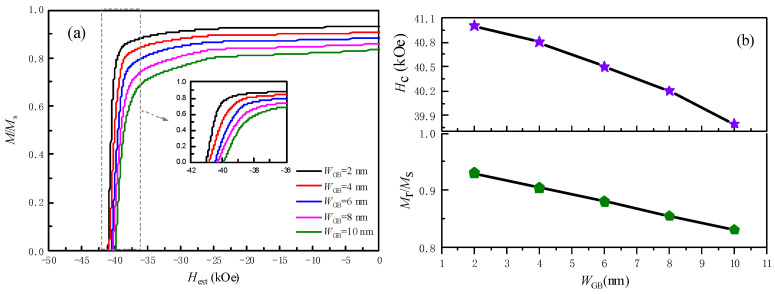
(**a**) Simulated demagnetization curves of nanoscale muti-grain Nd_2_Fe_14_B with different grain boundary widths, *W*_GB_, at 300K, with K = 4.5 × 10^6^ J/m^3^ and external field (*H*_ext_) ranging from −80 kOe to +80 kOe. (**b**) Coercivity (*H*_c_) and normalized remanence (*M_r_*/*M_s_*) versus *W*_GB_ of nanoscale muti-grain Nd_2_Fe_14_B magnet.

Figure 4 shows demagnetization curves for models with grain boundary width (*W*_GB_) ranging from 2 nm to 10 nm, referring to the parameters in Table 1. Both coercivity and remanence decrease monotonically as the *W*_GB_ increases.

For a *W*_GB_ of 2 nm, the coercivity is 42.3 kOe and *M_r_*/*M_s_* = 0.96. When the *W*_GB_ increases to 10 nm, *H_c_* drops to 28.7 kOe (a 32% decrease) and *M_r_*/*M_s_* falls to 0.81 (a 16% decrease). As the *W*_GB_ increases, the volume fraction of this magnetically dilute phase rises, thereby reducing the overall magnetization of the composite magnet. This effect directly accounts for the observed decline in remanence, since the contribution of the GB phase to the net magnetic moment is limited. Simultaneously, a wider GB introduces more magnetically weaker phases, where reverse domains can preferentially nucleate under an external field. These nucleation sites become more numerous and more effective as the *W*_GB_ expands. Once a reverse domain is nucleated, it can easily propagate into adjacent grains due to the reduced energy barrier for domain wall motion, leading to a lower coercive field.

**Figure 5:**
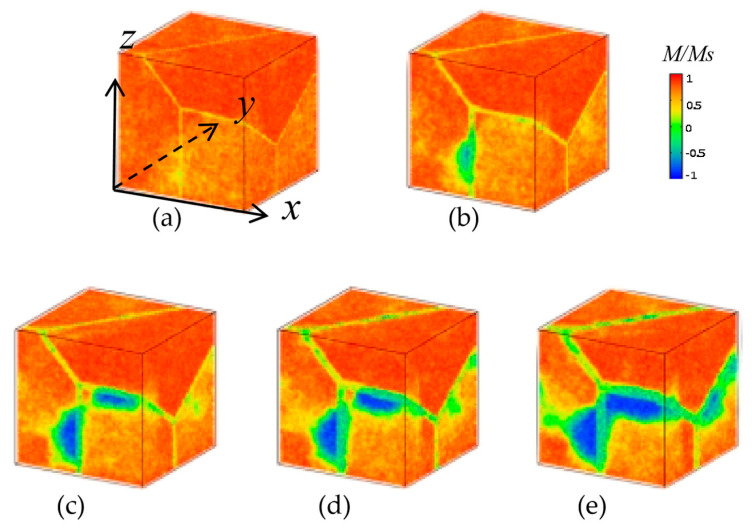
Magnetization reversal processes of muti-grain Nd_2_Fe_14_B magnets at *H*_ext_ = 36 kOe along the *z*-axis for GB widths of (**a**) 2 nm, (**b**) 4 nm, (**c**) 6 nm, (**d**) 8 nm, and (**e**) 10 nm.

Figure 5 presents the magnetization reversal process at an external field of *H_ext_* = 36 kOe. At *W*_GB_ = 2 nm, the sample remains mostly magnetized, with only minor local disturbances near GB regions. A narrow GB is insufficient to nucleate stable reverse domains, and the strong exchange coupling between grains helps maintain a uniform magnetization state. When W_GB_ increases to 4 nm, nucleation begins to occur preferentially at GB regions. The slightly wider GB provides a sufficient local reduction in magnetocrystalline anisotropy and exchange stiffness, allowing reverse magnetic moments to emerge under the applied field. However, at this stage, reverse domains remain confined near GB and do not yet propagate into grain interiors. For W_GB_ > 4 nm (i.e., 6 nm, 8 nm, and 10 nm), reverse domains have already formed and expanded into grain interiors. The wider GB acts as an effective nucleation site, and once a reverse domain is nucleated, the reduced energy barrier for domain wall motion allows it to grow rapidly into adjacent grains, leading to significant demagnetization.

A key observation is that nucleation consistently occurs at the GB edges, not at grain centers. Furthermore, domain walls are pinned at GBs during the reversal process. This dual behavior confirms that GBs serve simultaneously as both nucleation sites and pinning centers.

As nucleation sites, a wider GB facilitates the formation of reverse domains at lower external fields.

As pinning centers, GBs impede the motion of domain walls, which can temporarily delay full reversal, but in the presence of a wide GB, the pinning effect is insufficient to compensate for the enhanced nucleation.

### 3.2. Effect of Grain Boundary Magnetic Parameters

We discussed the influence of different GB magnetic parameters (i.e., *M_s_*′, *K*′, *A*_2_*) on coercivity and remanence under the same *W*_GB_ = 2 nm. Since the *W*_GB_ is relatively narrow and the proportion of the weakly magnetic phase is small, the variation ranges of both coercivity and remanence are weak, although the change trend can still be observed.

**Figure 6:**
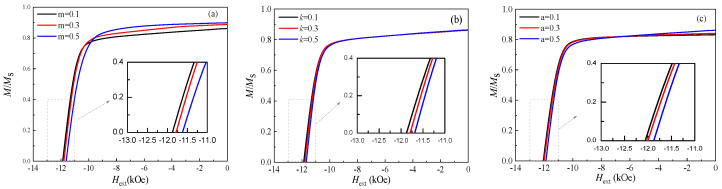
Effect of (**a**) *M_s_*′; (**b**) *K*′; and (**c**) *A*_2_* on the *H*_c_ and *M_r_*/*M*_s_, referring to the parameters in Table 1, with *N* = 6, K = 1 × 10^6^ J/m^3^, and *W*_GB_ = 2 nm.

Figure 6a shows how the GB saturation magnetization (*M_s_*′) affects magnetic properties. As *M_s_*′ increases from 0.1 *M_s_* to 0.5 *M_s_* (*m = M_s_*′*/M_s_* in Figure 6a), the remanence increases from 0.88 to 0.96, while coercivity was slightly reduced. This trade-off reflects two competing effects. Higher GB magnetization increases the overall *M_s_*, boosting remanence. But it also strengthens the exchange coupling between grains, making collective reversal easier and reducing coercivity. This explains the experimental observations that a non-magnetic GB—achieved by adding elements like Cu or Ga—yields higher coercivity [27,28].

Figure 6b shows that increasing the GB anisotropy *K*′ increases from 0.1 K to 0.5 K (*k = K*′*/K* in Figure 6b) raises coercivity from 11.7 kOe to 11.9 kOe, while remanence stays nearly constant. This result directly supports the GB diffusion strategy, where heavy rare earth elements like Dy form high-anisotropy shells around Nd_2_Fe_14_B grains. The enhanced anisotropy at the GB strengthens domain wall pinning and increases the nucleation field [12,29].

Increasing the GB exchange *A*_2_* from 0.1 A* to 0.5 A* (*a = A*_2_*/A* in Figure 6c) slightly reduces coercivity (from 12.1 kOe to 11.9 kOe) and slightly increases remanence (Figure 6c). Stronger exchange coupling across grains and the GB promotes magnetization reversal. Weakening this intergranular exchange coupling can enhance coercivity. Experimentally, it has been shown that adding elements with low solubility at sintering temperatures, such as Nb, Mo, or Zr, leads to the formation of precipitates within the Nd_2_Fe_14_B grains and modifies the GB chemistry. These microstructural changes are believed to reduce the intergranular exchange coupling, thereby contributing to an increase in coercivity [30].

### 3.3. Effect of Grain Crystallographic Orientation

**Figure 7:**
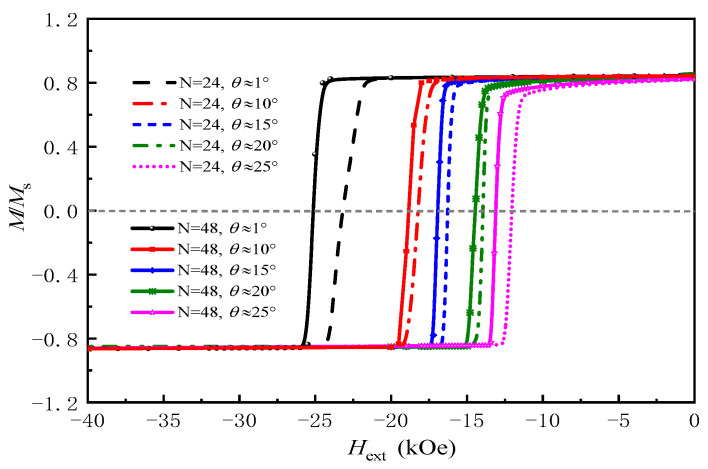
Demagnetization curves of Nd-Fe-B magnets with different easy-axis tilt angles for *N* = 24 and *N* = 48 with the parameters in Table 1: K = 2.1 × 10^6^ J/m^3^ and *W*_GB_ = 4 nm.

With fixed grain boundary parameters, we also investigated the effects of grain anisotropy orientation on coercivity and remanence. Figure 7 shows how the average easy-axis tilt angle affects demagnetization curves for models with 24 and 48 grains. As $\bar{\theta }$ increases from 2.6° to 11.6°, coercivity drops from 41.2 kOe to 31.5 kOe for *N* = 24, and from 44.8 kOe to 34.2 kOe for *N* = 48. In contrast, the remanence remains nearly constant across the entire range of tilt angles, indicating that the easy-axis misalignment primarily affects the coercive field rather than the saturation magnetization.

This behavior is consistent with previous experimental and theoretical work [31]. When the easy axes of the grains are well-aligned, their magnetocrystalline anisotropy fields act cooperatively, leading to a higher energy barrier for the nucleation of reverse domains and thus a larger *H_c_*. Conversely, an increase in misalignment disrupts this cooperative effect, reducing the effective anisotropy field and facilitating the nucleation of reverse domains, which results in a significant decrease in *H_c_*.

## 4. Conclusions

This work employed Hybrid Monte Carlo micromagnetic simulations and a Voronoi-tessellated polycrystalline model to systematically study the effects of grain boundary features and grain crystallographic orientation on the coercivity and remanence of Nd-Fe-B permanent magnets at 300 K. Our main findings are as follows:

Grain boundary width significantly impacts both coercivity and remanence. Increasing the *W*_GB_ from 2 nm to 10 nm reduces *H_c_* by 32% and *M_r_*/*M_s_* by 16%. The GB serves as both a nucleation site (wider GBs promote nucleation) and a pinning center (domain walls stop at the GB). The enhanced nucleation effect from a wider GB outweighs the pinning effect, leading to reduced coercivity.

GB magnetic parameters regulate coercivity and *M_r_* with distinct trends: higher GB saturation magnetization improves remanence but slightly reduces coercivity by strengthening intergranular exchange coupling. Higher GB anisotropy increases coercivity, as it strengthens domain wall pinning, supporting the Dy-diffusion strategy. A stronger GB exchange coupling slightly reduces coercivity due to promoted cooperative magnetization reversal.

Grain crystallographic orientation impacts coercivity: increasing the average easy-axis tilt angle from 2.6° to 11.6° reduces coercivity by about 24% for both 24 and 48-grain models. as misalignment weakens the cooperative anisotropy field and lowers the reverse domain nucleation energy barrier.

For high-coercivity Nd-Fe-B magnet design, a thin GB with low magnetization, high anisotropy and weak exchange coupling, combined with high-precision easy-axis alignment, are optimal. These findings provide quantitative theoretical guidance for the experimental preparation and microstructure modification of high-performance Nd-Fe-B permanent magnets.

## Figures and Tables

**Table 1:** Magnetic parameters of Nd_2_Fe_14_B at 300 K.

Inside grains	$M_{s}$ (A/m)	*K * (J/m^3^)	A* (J/m)
	1.281 × 10^6^	1~4.5 × 10^6^	1.25 × 10^−11^
At grain boundary	*M′_s_*/ Ms	*K′*/*K*	A*_2_/A*
	0.1~0.5	0.1~0.5	0.1~0.5
Other parameters	*W*_GB_ (nm)	*α_θ_*	*T* (K)
	2~10	32	300

**Table 2:** Average inclination angles $\bar{\theta }$ for different $\alpha_{\theta }$ and $c_{1}$ with number of grains is 24.

$\bar{\theta }$	$\alpha_{\theta }$	$c_{1}$
10°	11.6	0.057
15°	5.80	0.040
20°	3.80	0.031
25°	2.60	0.025

## Data Availability

The original contributions presented in this study are included in the article. Further inquiries can be directed to the corresponding author.
